# Supplementary material for: Formulation Development of Meloxicam Binary Ethosomal Hydrogel for Topical Delivery: In Vitro and In Vivo Assessment
Source: Pharmaceutics. 2024 Jul 4;16(7):898. doi: 10.3390/pharmaceutics16070898 (PMC11280089; doi:10.3390/pharmaceutics16070898)
Supplement: Supplementary file 1 [file pharmaceutics-16-00898-s001.zip › pharmaceutics-3033223-supplementary.pdf]

Box Behnken Design Expert Equations

The equations below constitute the polynomial model's actual equation for the response (vesicle size, dispersity index, EE%, and zeta potential) used to evaluate the variable's influence. The positive and negative coefficients refer to the variables' positive and negative impact on the response. where X1 is soya lecithin w/w%, X2 is ethanol w/w%, and X3 is propylene glycol w/w%.

Vesicle Size = $+210.69583 - 12.85833\ X1 - 8.43472\ X2 - 6.81958\ X3 + 0.352778\ X1X2 + 0.724167\ X1X3 + 0.278556\ X2X3 - 0.750000\ X1^2 + 0.249519\ X2^2 + 0.094083\ X3^2$

Dispersity Index = $+1.07281 - 0.205833\ X1 - 0.041751\ X2 - 0.011191\ X3 + 0.001058\ X1X2 - 0.000057\ X1X3 + 0.000425\ X2X3 + 0.030775\ X1^2 + 0.000657\ X2^2 + 0.000142\ X3^2$

EE% = $-341.83208 + 184.42865\ X1 + 10.42293\ X2 - 0.649142\ X3 - 0.953147X1X2 + 1.16953X1X3 - 0.033977X2X3 - 27.38734X1^2 - 0.126967X2^2 - 0.112661X^2$

Zeta Potential = $-65.01792 + 9.78542\ X1 + 0.965694\ X2 - 0.836250\ X3 - 0.383000\ X1X2 + 0.107917\ X1X3 + 0.008261\ X2X3$

The Optimized Formula Zeta Sizer Reading

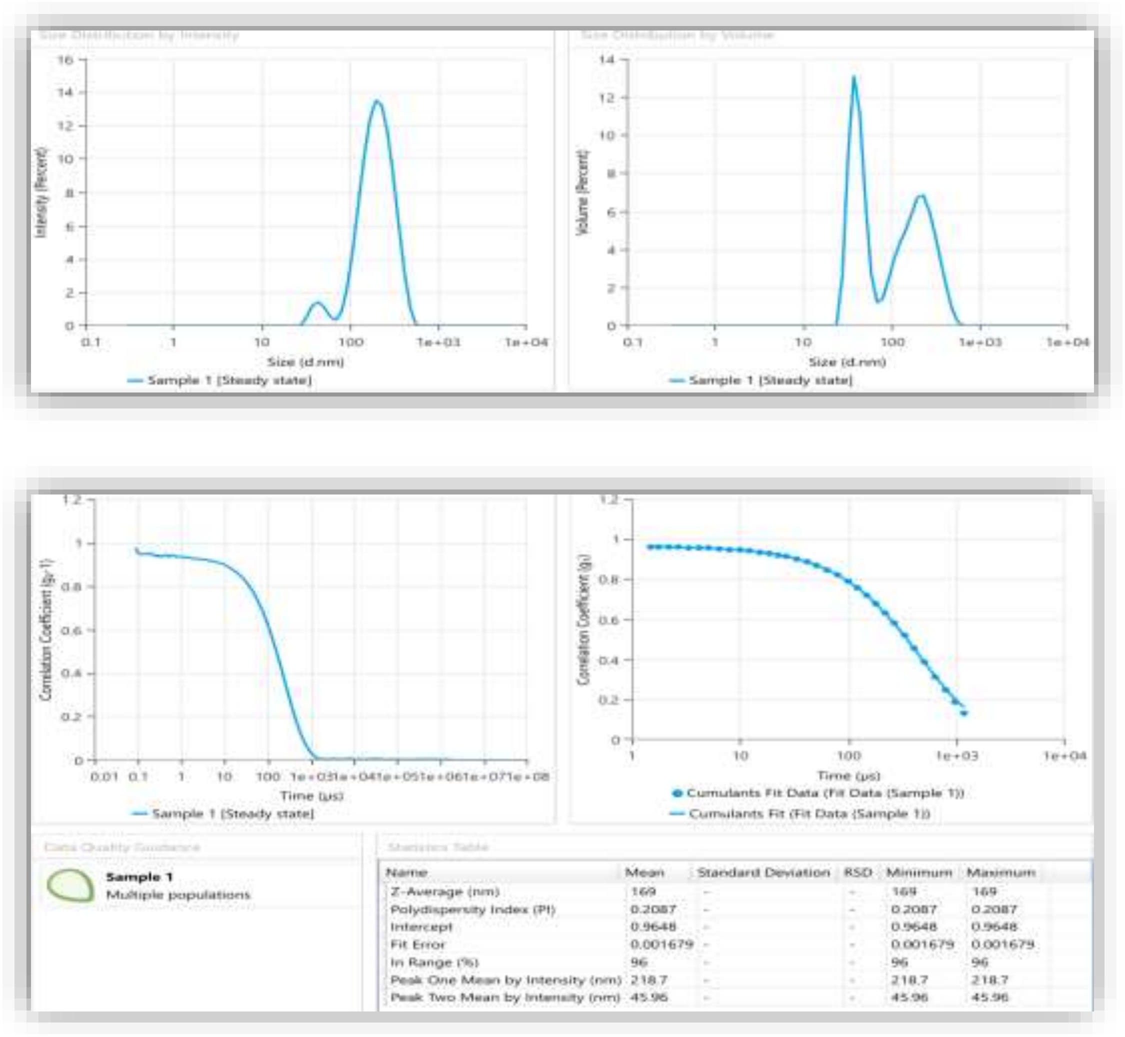

Figure S1. Vesicle size and dispersity index measurement of MLX-Ethos-OF

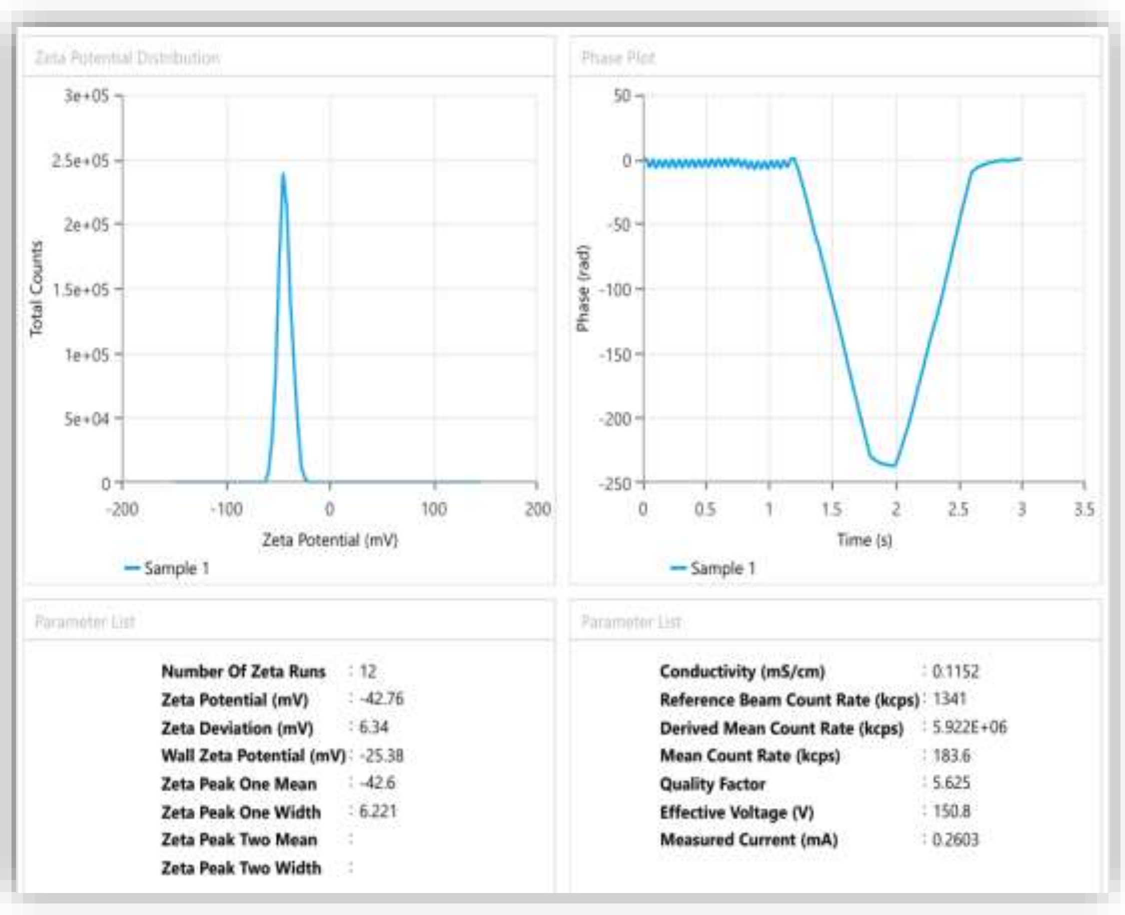

Figure S2. Zeta potential measurement peaks
